# Synthesis of nanoparticles of cobalt protoporphyrin IX (Co(iii)PPIX NPs). Antiradical, cytotoxicity and antibacterial properties

**DOI:** 10.1039/d5ra07110k

**Published:** 2025-10-03

**Authors:** Piotr Fijałkowski, Olga Impert, Paweł Pomastowski, Katarzyna Rafińska, Justyna Walczak-Skierska, Paweł Fijałkowski, Anna Katafias, Rudi van Eldik

**Affiliations:** a Centre for Modern Interdisciplinary Technologies, Nicolaus Copernicus University in Toruń Wileńska 4 87-100 Toruń Poland; b Department of Inorganic and Coordination Chemistry, Faculty of Chemistry, Nicolaus Copernicus University in Toruń Gagarina 7 87-100 Toruń Poland oimpert@umk.pl katafias@umk.pl; c Department of Environmental Chemistry and Bioanalytics, Faculty of Chemistry, Nicolaus Copernicus University in Toruń Gagarina 7 Toruń 87-100 Poland; d Department of Chemistry and Pharmacy, University of Erlangen-Nuremberg Egerlandstrasse 1 91058 Erlangen Germany

## Abstract

Porphyrins, composed of a tetrapyrrolic core and various substituents, are highly valued for their photo-, catalytic, electrochemical, and biochemical properties. Protoporphyrin IX (PPIX) is a heterocyclic organic compound whose porphyrin ring enables the chelation of transition metals, resulting in metalloporphyrins with diverse biological functions. Cobalt protoporphyrin IX (Co(iii)PPIX) is known as an inducer of heme oxygenase-1 (HO-1) and has been identified as a potential photosensitiser for use in photodynamic cancer therapy. In this study, Co(iii)PPIX nanoparticles (NPs) were synthesised *via* the antisolvent method. The resulting NPs were thoroughly characterised using FTIR spectroscopy, Raman spectroscopy, UV-Vis spectroscopy, DLS, SEM, and EDX techniques. Furthermore, their biological properties were investigated, including antibacterial activity (against *E. coli*, *S. aureus*, and *K. pneumoniae*), antioxidant activity, and cytotoxicity tests on Caco-2 and L929 cell lines. The antisolvent method allowed for the synthesis of NPs with a hydrodynamic size below 300 nm, and for one sample, below 200 nm, depending on the conditions. Following synthesis, ultrafiltration (UF) was employed to purify the samples by removing particles not involved in NP formation. A comparative study was conducted between samples subjected to UF and those that were not. The results indicate that using UF led to the synthesis of smaller NPs (307.7 *vs.* 190.2 nm), differences in Raman spectra, and modified biological properties.

## Abstract 2

Co(iii)PPIX nanoparticles were synthesized and their physicochemical properties were characterized. Furthermore, their biological properties were investigated, including antibacterial activity, antioxidant activity, and cytotoxicity test.

## Graphical abstract

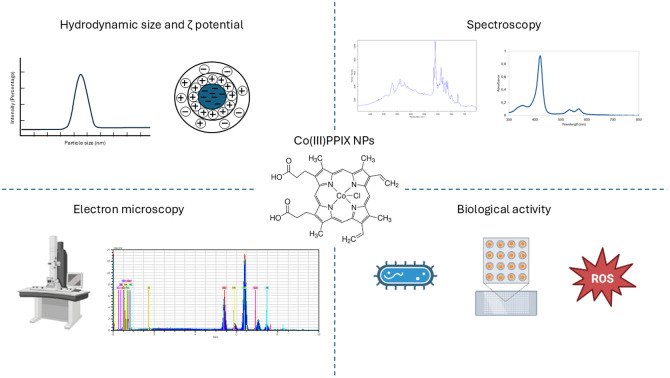

## Introduction

Porphyrins are organic compounds composed of a tetrapyrrolic macrocyclic core with various substituents. They are aromatic due to a conjugated system of 18 π electrons^1^ and can coordinate metal cations. Porphyrins have a wide range of photo-, catalytic-, electro-, and biochemical properties^2^ and are utilised in applications such as magnetic resonance imaging,^3,4^ cancer treatment,^5,6^ and catalysis.^7^

**Fig. 1:**
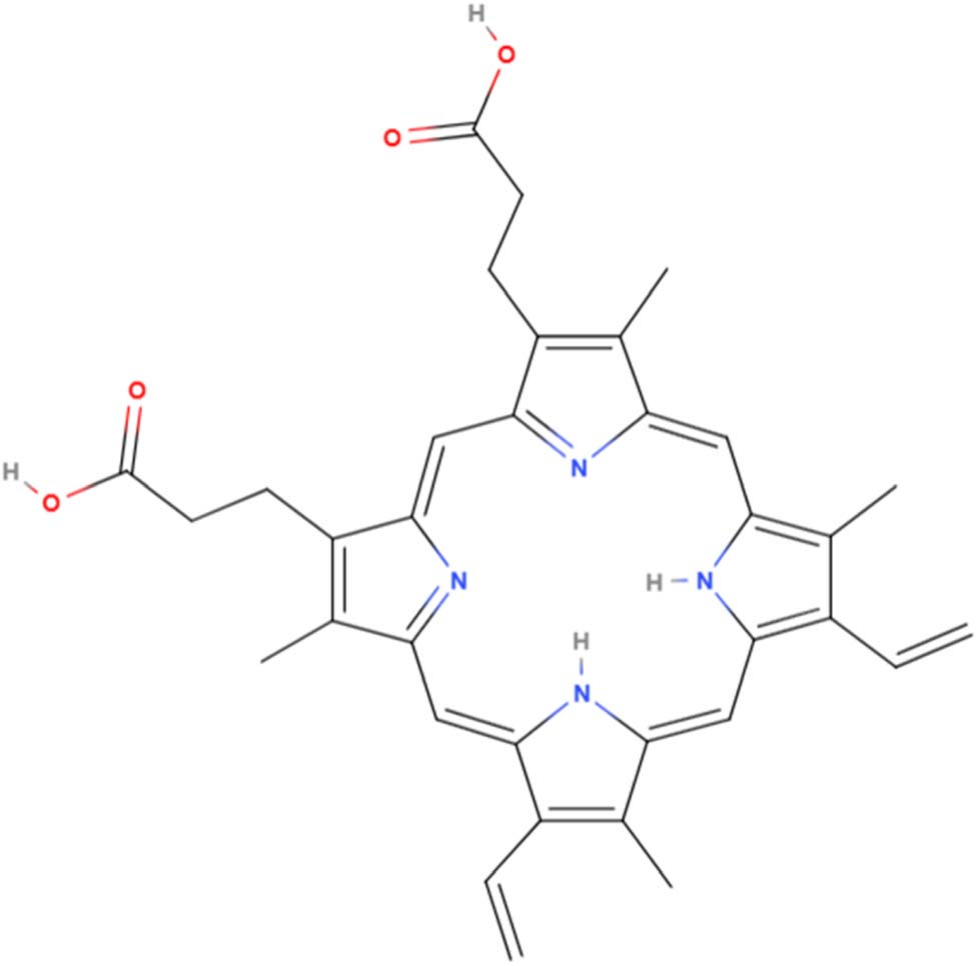
Molecular structure of protoporphyrin IX.

Protoporphyrin IX (PPIX) (Fig. 1) contains a porphyrin core with four methyl, two vinyl, and two propionic acid groups, allowing it to chelate transition metals. PPIX is a biosynthetic precursor of heme, chlorophyll, and cobalamin (vitamin B12) compounds. PPIX is the last intermediate product in the eight-step process of heme biosynthesis. Iron-substituted PPIX (heme) is a component of hemeproteins involved in oxygen and electron transport.^8^ PPIX generates reactive oxygen species (ROS) upon exposure to light, which induce cytotoxicity by damaging proteins, lipids, and DNA.^9^ Cobalt-substituted PPIX (CoPPIX) induces heme oxygenase-1 (HO-1) *via* the Nrf-2/HO-1 pathway.^10^ HO-1 degrades heme into bilirubin, carbon monoxide, and iron and plays a role in the oxidative stress response by preventing ROS formation,^11^ which may have an impact on the biological properties (cytotoxic, antibacterial, antiradical) of the studied nanoparticles. CoPPIX enhances cytokine expression related to granulocyte mobilisation.^12^ Nanomaterials exhibit promising multifunctional properties combining metal sensing with antibacterial and photocatalytic effects.^13–15^

PPIX nanoparticles have been extensively studied for photodynamic therapy in cancer treatment.^16,17^ PPIX NPs have also been investigated as antibacterial agents due to ROS production.^18^ Metal-chelated PPIX, such as ZnPPIX NPs, have been proposed as potential anticancer agents due to HO-1 inhibition.^19^ In contrast, CoPPIX is an inducer of HO-1, which may increase the survival of cell lines by protecting against ROS. CoPPIX NPs have also been explored for photoacoustic imaging.^20^ A polyethyleneimine-gold PPIX nanocomposite has been used as an electrochemical sensor for hydrogen peroxide detection.^21^ PPIX compounds are known for their photosensitising properties^22^ and are widely used as photosensitisers in photodynamic therapy. However, there is limited knowledge about the properties of PPIX compounds, particularly when complexed with cobalt, in nanometric systems.

Earlier, van Eldik and colleagues studied porphyrin complexes. This section briefly highlights the main results and conclusions from their work. The van Eldik group investigated the reactions of Fe(iii) porphyrin complexes. In one of their studies, it was established that water exchange in porphyrin complexes proceeds *via* a dissociative mechanism and that both the water exchange rate and the associated activation enthalpy depend on the porphyrin's charge.^23^ A series of studies was also conducted on nitric oxide (NO) binding to porphyrins. One key conclusion from this research is that the reversible binding of NO to heme proteins is governed by electronic factors (such as the pH of the solution) and structural factors (such as spin density reorganisation).^24^ Investigations involving other porphyrin systems confirmed the dissociative mechanism of reversible NO binding and the influence of porphyrin charge on the rate constants.^25–27^ Studies on cytochrome P450cam revealed that the kinetics of NO binding are significantly enhanced in the presence of a substrate (camphor), with the mechanism being dominated by bond formation.^28^ Research on NO binding to cobalamin, which contains a corrin ring (a reduced form of porphyrin), demonstrated that NO can bind rapidly and efficiently to the reduced form Cbl(ii) in a reversible manner.^29^ In contrast, no reaction occurs with aquacobalamin Cbl(H_2_O).^30^ Another line of research focused on the activation of hydrogen peroxide and O–O bond cleavage in model porphyrin systems and cytochrome P450. In this context, three transient intermediates were identified: ferric hydroperoxide (compound 0), the iron(iv)-oxo porphyrin π-cation radical (compound I), and iron(iv)-oxo (compound II).

Studies on O–O bond cleavage suggested that the environment's acidity significantly affects the nature of the resulting product and that the initial cleavage mechanism does not always correspond to the final reactive species observed under specific reaction conditions.^31,32^ The effect of the axial ligand, particularly *N*-methylimidazole, on the reactivity of high-valent iron(iv)-oxo species in heme enzyme models was also investigated. In contrast to compound I, both experimental and computational results indicated a lack of influence of the axial ligand on compound II.^33^ Furthermore, the van Eldik group provided the first evidence that, by suppressing heterolytic O–O bond cleavage, compound 0 can act as an oxygen donor, contributing to its catalytic activity.^34^ However, for colloidal, nanometric systems, this area remains unexplored. It indicates a gap in current knowledge regarding the detailed mechanism of action of oxygen donors in the form of CoPPIX NPs.

Due to the limited information available in the literature on PPIX nanoparticles, especially in combination with cobalt, we decided to synthesise cobalt protoporphyrin IX nanoparticles to study their physicochemical properties and biological activity. Therefore, this work aims to synthesise cobalt(iii) protoporphyrin IX nanoparticles (Co(iii)PPIX NPs) and investigate their physicochemical properties using DLS, UV-Vis, and Raman spectroscopy, SEM, and EDX. The biological properties of the synthesised Co(iii)PPIX NPs were also examined, including radical scavenging activity, cytotoxicity in cell lines, and antibacterial activity. Nanoparticle synthesis was performed using the antisolvent method, *i.e.*, by dissolving a given amount of the compound in a solvent and adding an appropriate volume of a miscible non-solvent in which the compound is insoluble. In this bottom-up nanoparticle synthesis process, precipitation is achieved by reducing the solubility of a dissolved substance in solution. Generally, the antisolvent precipitation process consists of three main stages: (i) nucleation, (ii) particle growth, and (iii) agglomeration, with supersaturation being the driving force for all three steps.^35^ This synthesis method was selected due to its rapidity, the ability to operate at ambient temperature and atmospheric pressure, and the lack of necessity for expensive equipment. The synthesis method used in this work complements earlier methods of silver nanoparticle synthesis.^36^ This study also applied ultrafiltration (UF) to remove particles not involved in nanoparticle formation, thereby providing dual optimisation in physicochemical properties and biological activity. Double optimisation has been reported, among others, by Wang *et al.* using the example of econazole salt cocrystal.^37^ To this end, a comparative study of physicochemical properties and biological activities was carried out between nanoparticle samples subjected to UF and those left untreated. The study of nanoparticles' biological properties was conducted similarly to the research by Zeng *et al.*, who also used methods based on bioactivity.^38^

## Experimental

### Materials and methods

All chemicals were of analytical reagent grade and used without further purification. Cobalt protoporphyrin IX chloride, absolute ethanol, molecular sieves, Amicon Ultra Centrifugal filters, sodium chloride, and 2,2-diphenyl-1-picrylhydrazyl radical were purchased from Sigma-Aldrich.

### Preparation of Co(iii)PPIX solution

A stock solution of Co(iii)PPIX (1 mg mL^−1^) was prepared in absolute ethanol (with molecular sieves to remove water). Dilutions were made to obtain 0.1, 0.01, and 0.001 mg mL^−1^. Six samples were prepared for each concentration with varying water (W) or saline (S) content (1, 2, 3, 10, 20, and 30% v/v) to induce Co(iii)PPIX nanoparticle precipitation.

### Ultrafiltration

Another set of samples was prepared as described above, but subjected to ultrafiltration (UF) using Amicon Ultra-4 tubes with a 3000 Da molecular weight cutoff. 4 mL of the solution was filtered by centrifuging using a Centrifuge 5810 R (Eppendorf) until half the volume was filtered. 2 mL of the matrix solution was then added, and centrifugation was repeated. The resulting retentate was used for further analysis.

### Hydrodynamic size and ζ (zeta) potential measurements

The hydrodynamic size of the nanoparticles was determined by dynamic light scattering (DLS) using a Zetasizer Nano ZS (Malvern Instruments). Two sets of samples (solutions and retentates) were transferred to measurement cuvettes, and each sample was measured in triplicate. ζ-potential measurements were performed under the same conditions. All measurements were taken at a scattering angle of 173°.

### UV-Vis spectroscopy

UV-Vis spectra were recorded at a concentration of 0.005 mg mL^−1^ by twofold diluting 0.01 mg mL^−1^ samples. Measurements were conducted in the 190–840 nm wavelength range using a NanoDrop 2000c spectrophotometer (Thermo Fisher Scientific, USA).

### Microscopic techniques

Seven samples were selected for further study based on preliminary results: (1) 0.1 mg mL^−1^ 2% S before UF, (2) 0.1 mg mL^−1^ 2% S after UF, (3) 0.01 mg mL^−1^ 1% S after UF, (4) 0.01 mg mL^−1^ 1% S before UF, (5) 0.01 mg mL^−1^ 1% water-added (W) before UF, (6) 0.01 mg mL^−1^ 1% W after UF, and (7) 0.1 mg mL^−1^ 20% S before UF. Imaging was performed on an SEM/FIB Quanta 3D FEG (FEI, Gräfelfing, Germany). Additionally, scanning electron microscopy (SEM, LEO 1430 VP, Leo Electron Microscopy Ltd, Cambridge, UK) and an energy-dispersive X-ray detector (EDX, Xflash 4010, Bruker AXS, Bremen, Germany) were used. HR-SEM was conducted in secondary electron (SE) mode, while SEM-EDX was performed in backscattered electron (BSE) mode.

### Raman spectroscopy

The seven samples prepared for HR-SEM were applied to H17 stainless steel plates by depositing 15 μL of solution thrice per spot. Measurements were acquired using a SENTERRA II spectrometer (Bruker Optik, Germany) in the 400–4000 cm^−1^ range. Data were processed using OPUS 7.5 software (Bruker Daltonics, Hamburg, Germany).

### Fourier transform infrared spectroscopy

Sample preparation for FTIR analysis was carried out in the same manner as for Raman spectroscopy. The study was performed using a vacuum FTIR spectrometer Vertex 70V, equipped with a Hyperion 1000 microscope (Bruker Optik, Germany). Data were processed with OPUS 7.5 software (Bruker Daltonics, Hamburg, Germany).

### Radical scavenging activity

Radical scavenging activity of the seven selected samples was assessed using the DPPH assay. 50 μL of each sample was placed in a 96-well plate in triplicate. Subsequently, 200 μL of DPPH (0.1 M in methanol) was added to each well, and the plate was incubated in the dark for 30 min. Absorbance was measured at 517 nm using a VarioSkan Lux spectrophotometer (Thermo Scientific). Antioxidant activity was calculated using the following equation:
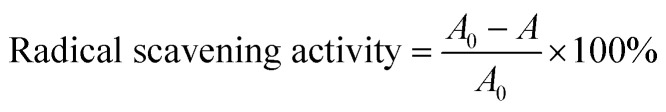
where: *A*_0_ – mean absorbance of the control. *A* – mean absorbance of the sample.

### Cytotoxicity

An MTT assay was performed to evaluate the cytotoxicity of the synthesised Co(iii)PPIX NPs. The assay was conducted on two cell lines, Caco-2 and L929, which were seeded into 96-well plates and incubated for 48 h. Afterwards, the seven selected samples were added at concentrations of 0.01, 0.005, 0.0025, 0.00125, 0.000625, 0.000313, 0.000267, and 0.0000781 mg mL^−1^, dissolved in DMSO. The control consisted of culture medium containing DMSO at matching concentrations. Following a 48 h incubation, 10 μL of MTT solution (5 mg mL^−1^ in PBS) was added to each well and incubated for an additional 4 h. After medium removal, 100 μL of DMSO was added, and absorbance was recorded at 560 nm using a VarioSkan LUX spectrophotometer (Thermo Fisher Scientific). Cell viability was expressed relative to the control (100%).

### Antibacterial activity

Three bacterial strains were selected for the antibacterial activity assay: *Escherichia coli*, *Staphylococcus aureus*, and *Klebsiella pneumoniae*. Bacterial suspensions were prepared to an optical density of 0.5 McFarland and then diluted 100-fold. A 96-well plate was filled with 190 μL of bacterial suspension (triplicate for each strain) and 10 μL of the test samples. The control group consisted of a bacterial suspension with ethanol, while the negative control contained bacterial medium with the test samples. Plates were incubated for 24 h at 37 °C. The following day, 12 μL of resazurin solution (100 μg mL^−1^) was added to each well and incubated for one hour at 37 °C. Antibacterial activity was assessed by measuring fluorescence using a microplate reader (Multiskan, ThermoFisher) with excitation at 560 nm and emission at 590 nm.

### High-performance liquid chromatography

The analysis of cobalt protoporphyrin IX (Co(iii)PPIX) nanoparticles in permeate and retentate fractions was performed using a Shimadzu LC-MS/MS 8050 system (Kyoto, Japan) with a C18 column (100 × 2.1 mm, 2.6 μm) and LabSolutions 5.8 software. Separation was achieved with a gradient elution of acetonitrile and formic acid in water (0.01–5.00 min, 20–100% ACN; 5–5.5 min, 100–20% ACN) at a flow rate of 0.3 mL min^−1^ and an injection volume of 8 μL. Detection was performed in positive ionisation modes using a triple quadrupole equipped with an ESI source (*m*/*z* 100–1300) under the following conditions: nebulising gas flow 3 L min^−1^, heating gas flow 10 L min^−1^, interface temperature 300 °C, and DL temperature 230 °C. Quantification was carried out in scheduled multiple reaction monitoring (MRM) mode. The MRM transitions for Co(iii)PPIX were: 619.1 → 560.15 with a collision energy of −40 eV.

Working solutions were prepared by diluting stock solutions with methanol to concentrations between 0.25–100 μg mL^−1^ and stored at −20 °C. Calibration curves were constructed, and limits of detection (LOD) and quantification (LOQ) were established at signal-to-noise ratios of 3 and 10, respectively. All measurements were performed in triplicate, and results are expressed as mean ± standard deviation. The LOD and LOQ were 0.05 and 0.165 μg mL^−1^, respectively.

## Results and discussion

### Hydrodynamic size and zeta potential

**Fig. 2:**
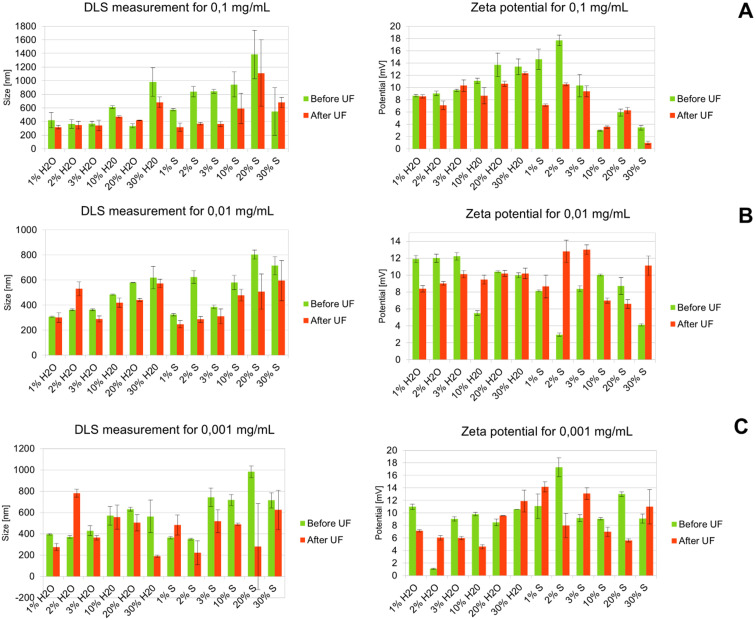
Results of hydrodynamic size and zeta potential DLS measurements for Co(iii)PPIX nanoparticles, where concetration of Co(iii)PPIX at 0,1 mg mL^−1^ (A), 0,01 mg mL^−1^ (B) and 0,001 mg mL^−1^ (C) .

Dynamic light scattering (DLS) is a technique that enables the measurement of the hydrodynamic diameter of particles ranging from 0.3 to 10 000 nm. The technique is based on fluctuations in scattered light intensity due to Brownian motion of suspended particles. Fig. 2 summarises the size and zeta potential measurements.

At 0.1 mg mL^−1^ (Fig. 2A), the smallest hydrodynamic diameter (316.4 nm) was observed for the 1% S UF-treated sample. Particle sizes below 400 nm were also found for 1–3% W and S. Except for 30% S, UF consistently reduced particle size. The largest diameter (1385 nm) was recorded for the 20% S. A correlation was found between the amount of antisolvent added and the hydrodynamic size; minor additions resulted in smaller particle sizes, and UF generally reduced sizes. The highest value (17.7 mV) for zeta potential was recorded for 2% S before UF. Elevated zeta potentials were observed for samples with high water content (20–30%) and low salt content (1–2%), whereas lower values were associated with higher salt content (10–30%). Non-UF samples generally exhibited higher zeta potentials.

At 0.01 mg mL^−1^ (Fig. 2B), the size distribution pattern was similar to that at 0.1 mg mL^−1^. The smallest size (248.2 nm) was recorded for 1% S, about 70 nm smaller than the lowest size at the higher concentration. Small sizes corresponded to lower antisolvent additions (1%, 2%, and 3%). The largest particles (803.7 nm) were observed for 20% S without UF, 581 nm smaller than at 0.1 mg mL^−1^. In this case, only one sample (2% W) had a smaller size without UF. Zeta potential was lower overall than at 0.1 mg mL^−1^. The highest zeta potential (13 mV) was recorded for 3% S after UF. Water-added samples had higher zeta potentials without UF, while salt-added samples had higher zeta potentials post-UF. As with 0.1 mg mL^−1^, W samples had higher average zeta potentials than S.

At 0.001 mg mL^−1^ (Fig. 2C), the smallest size (190.2 nm) was obtained for the 30% W sample. Smaller sizes were observed at higher concentrations for samples with low antisolvent additions. The largest size was again observed for 20% S. Post-UF size reduction was observed in only two cases (2% W and 1% S). The highest zeta potential (17.3 mV) was obtained for 2% S without UF, the highest across all samples.

Across all conditions, the largest hydrodynamic diameters were consistently observed at the 0.1 mg mL^−1^ concentration, with the 20% S sample having the most prominent sizes at all concentrations. Interestingly, the smallest particles were not always obtained at the lowest concentration (0.001 mg mL^−1^) but at 0.01 mg mL^−1^, where 9 of 12 non-UF-treated and 7 of 12 UF-treated samples exhibited the smallest hydrodynamic sizes. UF generally reduced particle size, supporting its role in removing free molecules that otherwise contribute to aggregation. For zeta potential, non-UF-treated samples showed the highest values in 6 of 12 cases at 0.1 mg mL^−1^, whereas among UF-treated samples, maximum values were observed in 6 of 12 cases at 0.01 mg mL^−1^.

The concentration of Co(iii)PPIX significantly affects the hydrodynamic size of the particles. The largest sizes were observed for samples with the highest tested concentration (0.1 mg mL^−1^). However, the smallest particles were not obtained at the lowest concentration but at 0.01 mg mL^−1^, whereas the smallest size was recorded at 0.001 mg mL^−1^ in general. The concentration of the substance may influence the hydrodynamic size since increasing concentration reduces intermolecular distances, leading to enhanced attractive forces and aggregation potential.^39^ The type of antisolvent also played a key role. Salt-containing samples generally formed larger particles with lower zeta potential values. These results may be accounted for by electrostatic screening effects promoting particle aggregation.^40,41^ Increasing ion concentration shields the repulsive electrostatic interaction between nanoparticles, decreasing surface hydration and consequently destabilising the dispersion.

Furthermore, adding a neutral electrolyte necessitates the adsorption of ions on the particle surface to maintain a constant surface potential, consequently causing a decrease in the zeta potential.^42^ A specific correlation between hydrodynamic size and zeta potential is most apparent in samples with added physiological saline. Although size and zeta potential measurements are not directly related (the standard practice for determining size is Stokes–Einstein's equation, which neglects the effect of the diffuse layer), a correlation between these two parameters can be observed. Since the zeta potential indicates the charge between a solid body and the liquid in which it is dispersed, a higher potential results in better particle stability in a given medium, thereby reducing their aggregation capability.

### UV-Vis spectroscopy

**Fig. 3:**
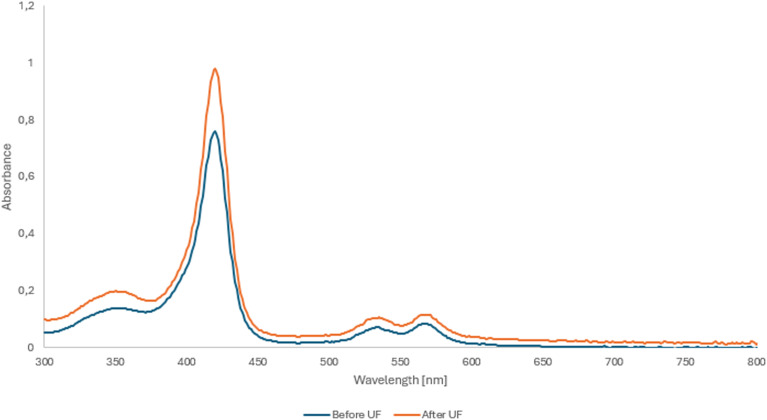
UV-Vis spectra of samples with 20% saline before and after ultrafiltration.

UV-Vis spectroscopy studies were performed for all samples, considering the amount of antisolvent added (water and salt), and for samples before and after UF, using a concentration of 0.005 mg mL^−1^. Fig. 3 shows a representative UV-Vis spectrum of Co(iii)PPIX nanoparticles, consisting of five absorption bands at 230, 354, 420, 534, and 568 nm, with the most characteristic one at 420 nm. UF-treated samples exhibited higher spectral intensities than non-UF samples, with exceptions observed for 2% W, 1% S, and 3% S. The most significant differences were noted between the 20% W and 30% W pairs.

The UV-Vis spectrum of porphyrins consists of two types of bands. The first, known as the Soret band, is a high-intensity band occurring at a wavelength of approximately 400 nm. The second type consists of Q bands, which have noticeably lower intensity and appear in the wavelength range of 500–600 nm. For free PPIX in ethanol, the Soret band appears at 402 nm, accompanied by four Q bands: Q1y (503 nm), Q0y (537 nm), Q1x (575 nm), and Q0x (629 nm).^43^ Its coordination with cobalt(iii) results in a considerable change in the electronic spectrum.

The Soret band appears at 420 nm, and the number of Q bands is reduced to two, occurring at 534 (*Q*_1_) and 568 (*Q*_0_) nm. The Soret band is due to the transition of electrons from the ground electron state to the second electron excited state. The *Q*_0_ band in metalloporphyrins results from the electron transition from the electron ground state to the first excited electron state (without molecular vibration). In contrast, the *Q*_1_ band is due to the same transition described earlier, but molecular vibrations are also involved.^44^ The reduction in the number of Q bands is attributed to the increased symmetry of the porphyrin upon metal coordination and the enhanced degeneracy of electron energy levels.^43,45^ In addition to these characteristic bands, our spectra revealed a minor absorption feature at 353 nm. It can be attributed to adding water or an aqueous NaCl to the samples, since PPIX in water is reported to display a Soret band at approximately 352 nm.^5^

In most cases, applying the UF process contributed to increased intensity of the absorption bands, although no shifts were observed. This effect may be attributed to reduced attenuation resulting from the filtration of salts and particles not involved in forming Co(iii)PPIX NPs.

### Electron microscopy

Scanning electron microscopy (SEM) is a powerful tool for analysing nanomaterials and nanoparticles, providing information on morphology, distribution, and composition without damaging the samples.^46^ Seven samples (SP1–SP7) were selected for further analysis based on hydrodynamic size and zeta potential. Samples of the finest particles and highest zeta potentials in saline and water were chosen in pairs (before and after UF), and one of the largest particles without UF. The samples included SP1 – 0.1 mg mL^−1^ 2% S without UF, SP2 – 0.1 mg mL^−1^ UF, SP3 – 0.01 mg mL^−1^ 1% S UF, SP4 – 0.01 mg mL^−1^ 1% S without UF, SP5 – 0.01 mg mL^−1^ 1% W without UF, SP6 – 0.01 mg mL^−1^ 1% W UF, and SP7 – 0.1 mg mL^−1^ 20% S without UF. SEM-EDX spectra confirmed the presence of cobalt, carbon, nitrogen, and oxygen—key elements in the Co(iii)PPIX structure. For the selected samples, the average cobalt mass content ranged from 0.87% to 1.02%, and the atomic content ranged from 0.75% to 0.87%. For the other elements in Co(iii)PPIX, the overall average mass and atomic contents were C – 1.25% and 5.17%, N – 1.84% and 6.48%, and O – 0.19% and 0.6%, respectively. The amounts of these elements, both in mass and atomic percentages, were low due to the low concentrations of Co(iii)PPIX used for the samples. Other observed elements, such as Fe, Cr, Ni, Mn, and Si, originated from the steel substrate on which the samples were deposited and from the stage used for analyses. Sodium and chlorine peaks were observed in SP3 and SP7, attributable to the NaCl antisolvent, with SP7 (20% S) showing the highest NaCl content due to its higher salt concentration.

**Fig. 4:**
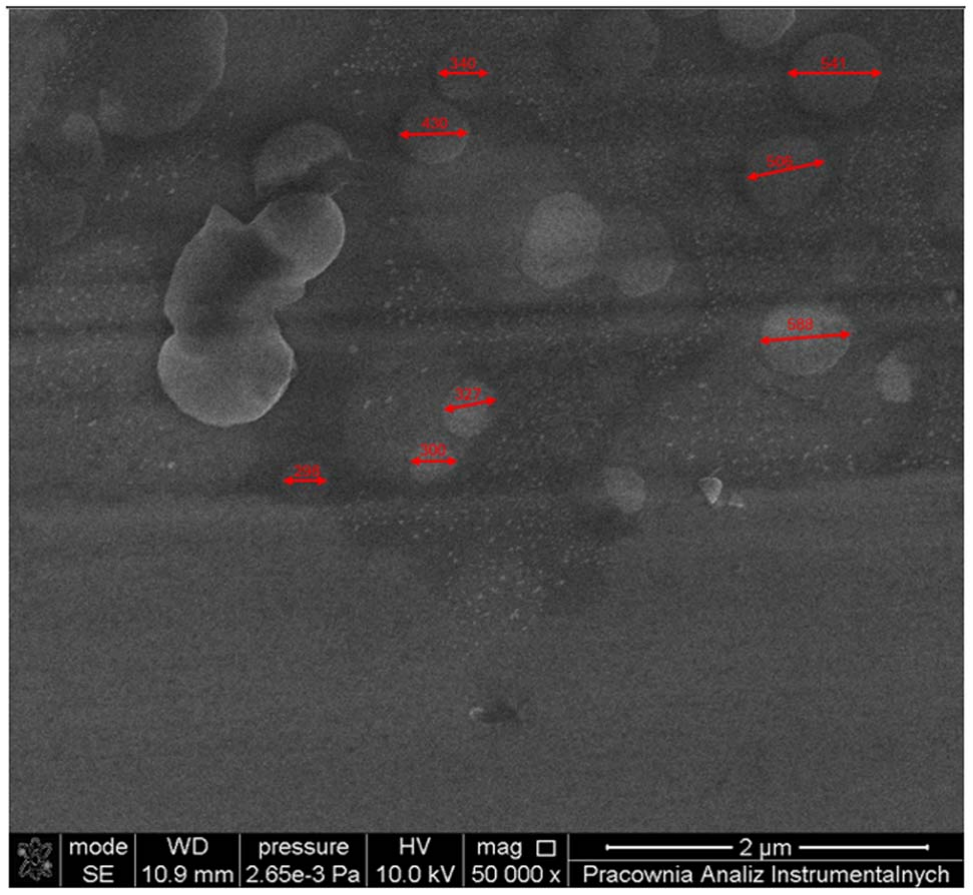
HR-SEM image of CoPPIX nanoparticles for SP2.

Table 1 summarises the average elemental mass and atomic content for all samples. A representative HR-SEM image of SP2 is shown in Fig. 4, where spherical nanoparticles are clearly visible, with diameters ranging from ∼230 to 560 nm.

**Table 1:** Average mass (atomic) content of elements in analysed samples by SEM-EDX

Sample	Element [%]										
	C	N	O	Si	Cr	Mn	Fe	Co	Ni	Na	Cl
1	1.29 (5.39)	1.60 (5.72)	0.15 (0.48)	0.45 (0.81)	16.95 (16.38)	1.80 (1.64)	68.80 (61.90)	1.02 (0.87)	7.94 (6.80)	—	—
2	1.29 (5.35)	1.95 (6.91)	0.19 (0.60)	0.48 (0.86)	17.03 (16.28)	1.70 (1.54)	68.72 (61.16)	0.92 (0.77)	7.71 (6.53)	—	—
3	1.30 (5.38)	1.88 (6.97)	0.21 (0.63)	0.50 (0.89)	17.04 (16.32)	1.74 (1.58)	68.82 (61.34)	0.89 (0.75)	7.62 (6.46)	1.75 (3.38)	0.32 (0.40)
4	1.23 (5.17)	1.60 (5.73)	0.16 (0.51)	0.47 (0.83)	17.03 (16.48)	1.82 (1.67)	68.83 (62.02)	1.01 (0.86)	7.85 (6.73)	—	—
5	1.19 (4.99)	1.74 (6.23)	0.21 (0.65)	0.46 (0.83)	16.84 (16.23)	1.73 (1.58)	69.13 (62.06)	0.98 (0.84)	7.72 (6.59)	—	—
6	1.40 (5.72)	2.17 (7.60)	0.20 (0.61)	0.45 (0.78)	17.16 (16.26)	1.67 (1.50)	68.47 (60.40)	0.95 (0.79)	7.54 (6.33)	—	—
7	1.06 (4.18)	1.93 (6.18)	0.22 (0.73)	0.36 (0.66)	14.53 (14.55)	1.63 (1.42)	59.42 (56.33)	0.87 (0.80)	6.62 (6.03)	3.82 (7.80)	1.00 (1.32)
Mean value	1.25 (5.17)	1.84 (6.48)	0.19 (0.60)	0.45 (0.81)	16.65 (16.07)	1.73 (1.56)	67.46 (60.74)	0.95 (0.81)	7.57 (6.50)		

### Raman spectroscopy

Raman spectroscopy analysis was conducted on seven selected samples (SP1–SP7) using two substrates: calcium fluoride (CaF_2_) and a steel plate. The porphyrin ring, consisting of a tetrapyrrole macrocycle, exhibits stretching vibrations of C–C and C–N bonds in the 1300–1700 cm^−1^ range, and for heme, they depend on the oxidation state of the iron atom. Raman shifts are observed at 1320, 1390, and 1579 cm^−1^ for the monomer pyrrole.^47^ This study detected corresponding signals at 1305.63, 1395.71, and 1591.47 cm^−1^, consistent with C–C bond vibrations within the pyrrole ring. The bands around 1580 cm^−1^ correspond to the so-called G bands, indicating C–C bond vibrations in the plane from the sp^2^ orbital.^48^ The obtained spectra closely matched previously reported Raman data for Co(iii)PPIX,^49^ which showed bands at 750, 1129, 1241, 1310, 1372, 1585, and 1642 cm^−1^, while in our case, they were 751, 1125, 1231, 1305, 1372, 1591, and 1642 cm^−1^. Higher wavenumber signals in the 2700–3000 cm^−1^ range, such as 2715 and 2892 cm^−1^, may correspond to stretching vibrations of CH_2_ and CH_3_ groups attached to the tetrapyrrole ring, while bands above 3000 cm^−1^ (3068 and 3182 cm^−1^) correspond to aromatic C–H stretching. Substrate-dependent differences were observed. On the steel substrate, stronger signals were obtained in the 2500–3200 cm^−1^ range, corresponding to CH_2_ and CH_3_ vibrations, suggesting that steel provides enhanced analytical conditions for these functional groups.

**Fig. 5:**
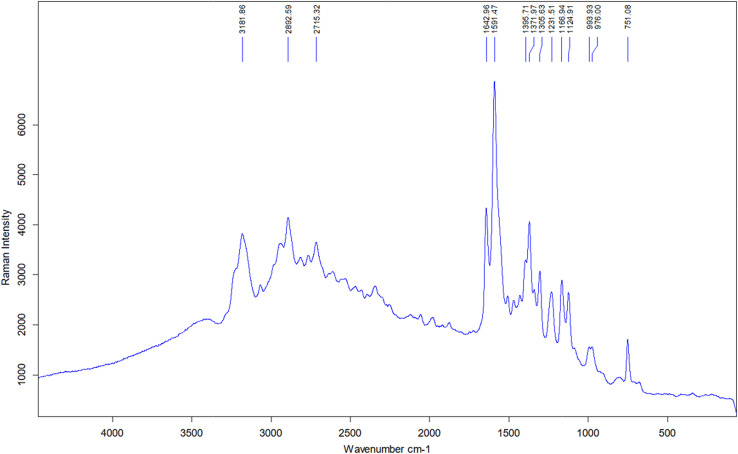
Raman spectrum obtained for SP1 on a steel substrate.

Significant spectra were recorded for SP1, SP2, and SP7 on CaF_2_, while SP3–SP5 showed no relevant signals. On the steel substrate, relevant signals were obtained for SP1 (Fig. 5) and SP7, with SP2-SP6 showing no significant signals. SP1 on steel showed two prominent signal ranges: 1000–1600 cm^−1^ and 2400–3400 cm^−1^.

**Fig. 6:**
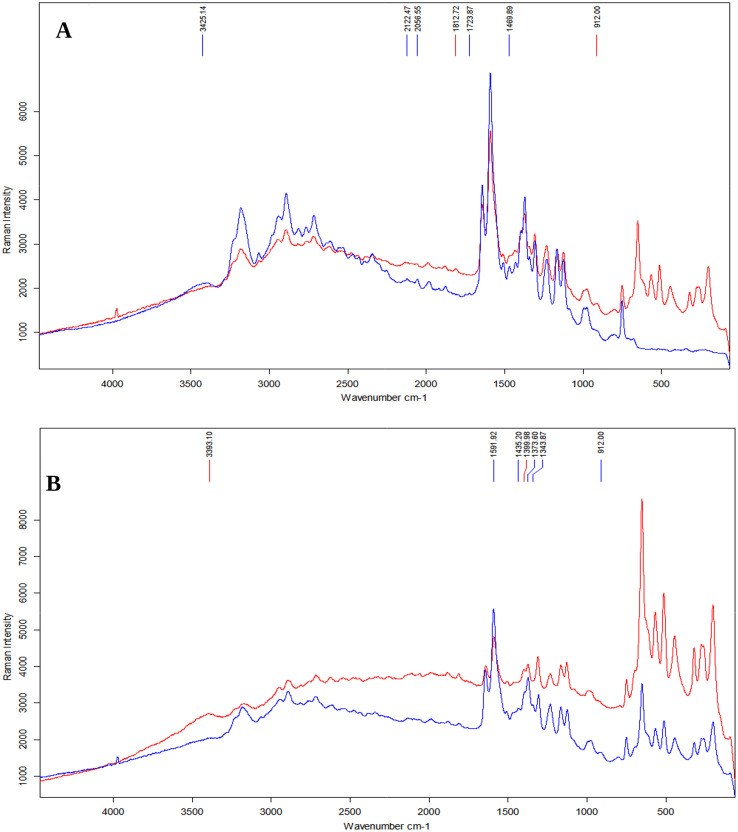
(A) – Comparison of the substrate's influence on the Raman spectroscopy spectrum for SP1. Blue represents the spectrum for the steel plate, while red represents the spectrum for CaF_2_. (B) – Comparison of the effect of the UF process on the spectra of SP1 and SP2 on the CaF_2_ substrate. Blue indicates SP1 (without UF), while red suggests SP2 (UF).

Comparing SP1 spectra from different substrates revealed differences (Fig. 6A). Signals in the 200–650 cm^−1^ for the sample on the CaF_2_ substrate were excluded, as these signals originate directly from the substrate itself. Additional bands at 1470, 1724, 2057, and 2122 cm^−1^, along with a broad band near 3400 cm^−1^, were present on steel, while CaF_2_ showed extra signals at 912, 1813, and 3973 cm^−1^. Signal intensity was higher on steel at 1592, 1643, and 2533–3219 cm^−1^, while CaF_2_ exhibited stronger peaks between 1125–1308 cm^−1^ and 1725–2344 cm^−1^.

Fig. 6B compares SP1 and SP2, illustrating the influence of UF on the Raman spectrum. SP2 had significantly higher overall intensity, except for the 1592 cm^−1^ signal, which was stronger in SP1. SP1 also showed additional signals (912, 1344, 1435, and 3973 cm^−1^) absent in SP2. Conversely, SP2 had a 1400 cm^−1^ signal and a broad band near 3400 cm^−1^, not present in SP1.

### Fourier transform infrared spectroscopy

**Fig. 7:**
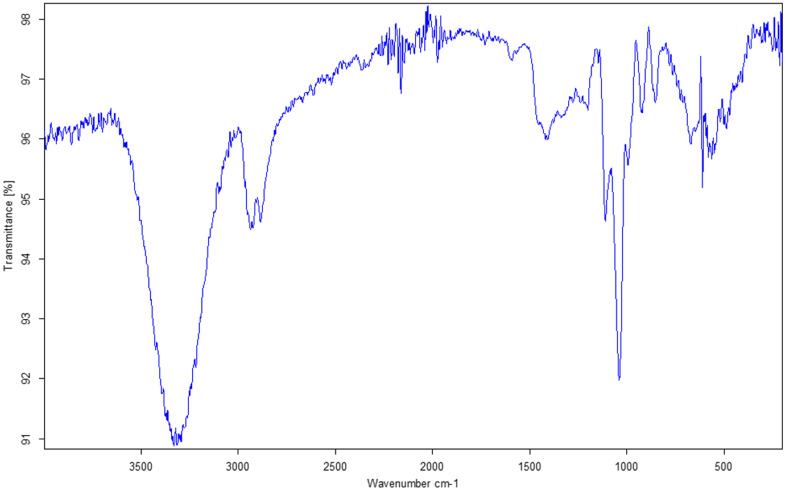
FTIR spectrum obtained for SP2 on CaF2 substrate.

FTIR measurements were performed using a CaF_2_ substrate. A significant analytical spectrum was obtained only for SP2 (Fig. 7). The broad band in the range of 3600–3000 cm^−1^ corresponds to the vibrations of N–H bonds present in the amine group and O–H bonds found in propionic acid residues. Bands in the 3000–2800 cm^−1^ range are attributed to C–H stretching in methyl and methylene groups. A small band at 1730 cm^−1^ corresponds to C=O bonds in carboxyl groups. The band at 1597 cm^−1^ is associated with the vibrations of C=C bonds within the porphyrin ring, while the signal at 1417 cm^−1^ is associated with aromatic C=C vibrations. The band around 1200 cm^−1^ originates from C–N stretching within the macrocycle,^50^ and additional signals at 1100 and 1039 cm^−1^ may also be linked to porphyrin ring vibrations. Fe–N bond vibrations are typically found at approximately 1000 cm^−1^ (ref. 51) in iron–porphyrin complexes. Thus, our study's signal at 994 cm^−1^ may originate from the Co–N bond. The signal at 854 cm^−1^ corresponds to bending vibrations of C–H and N–H.^50^ The bands in the 700–500 cm^−1^ range may be associated with chlorides, originating from the physiological saline used as an antisolvent for this sample (SP2).

### Antioxidant properties

The DPPH (2,2-diphenyl-1-picrylhydrazyl) assay is a simple, rapid, and cost-effective method for assessing the free radical-scavenging abilities of a compound. DPPH is a free radical with maximum absorbance at 517 nm. Upon reaction with an antioxidant, it changes from purple to yellow, thereby decreasing absorbance. The reduction in absorbance is proportional to the antiradical activity of the studied compound. Free radical removal can occur through electron transfer or hydrogen atom transfer. PPIX exhibits good free radical scavenging properties *via* the electron transfer mechanism; in contrast, the hydrogen atom transfer mechanism is not favourable for PPIX.^52^

**Fig. 8:**
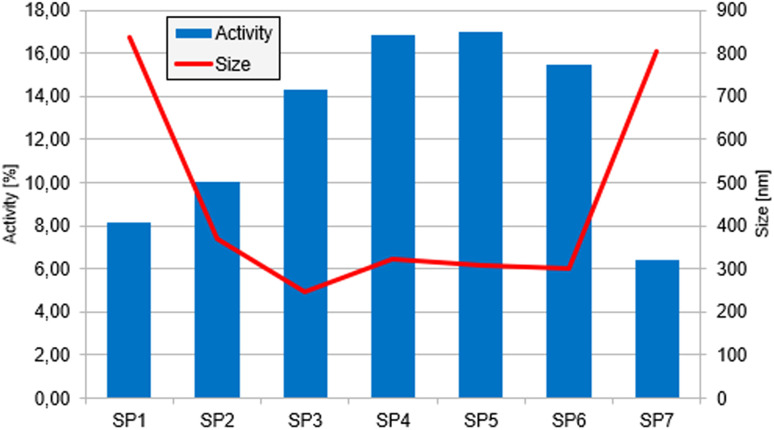
Results of the radical scavenging activity of Co(iii)PPIX NPs obtained from the DPPH test. The columns represent the radical scavenging activity, while the line illustrates the average hydrodynamic size of the individual samples.

The results of the DPPH assay for the seven selected samples are presented in Fig. 8. Co(iii)PPIX nanoparticles exhibit antioxidant properties, with activity ranging from 6.45% to 17.02%. The highest antioxidant activities were observed for SP4 and SP5 (16.85% and 17.02%), while the lowest were recorded for SP1 and SP7 (8.18% and 6.45%).

The results indicate a correlation between the size of Co(iii)PPIX nanoparticles and DPPH radical scavenging activity, with smaller sizes generally exhibiting better activity. This effect can be attributed to the smaller particles' increased surface area-to-volume ratio, providing more active sites for interaction with DPPH radicals.^53^ It is expected that a higher concentration of the studied compound would increase free radical scavenging activity; however, in our study, samples with lower concentrations exhibited the best activity. The relationship between size and activity may account for this phenomenon, as the significant size differences among samples mitigate the influence of Co(iii)PPIX concentration.

### Cytotoxicity profiles of Co(iii)PPIX nanoparticles

Cytotoxicity studies were conducted using two cell lines: Caco-2 and L929 (SI Fig. S1). Caco-2 cells were selected due to their well-established role as an *in vitro* model of the human intestinal epithelium. Upon differentiation, Caco-2 cells form a polarised monolayer with tight junctions, microvilli, and characteristic transporters, closely mimicking small intestinal enterocytes' physiological and functional properties. It makes them particularly suitable for assessing the intestinal absorption, permeability, and cytotoxicity of orally administered or gastrointestinally exposed nanoparticles. Given the potential biomedical applications of Co(iii)PPIX nanoparticles, particularly where oral exposure or systemic absorption through the gut is possible, it was essential to evaluate their interaction with intestinal epithelial cells. Caco-2 cells can provide a reproducible and physiologically relevant system for predicting the biocompatibility and safety profile of cobalt protoporphyrin IX nanoparticles upon intestinal exposure.

The L929 cell line was used as a non-tumorigenic reference model to evaluate the general cytotoxicity and biocompatibility of Co(iii)PPIX nanoparticles. L929 is a well-characterised murine fibroblast cell line derived from subcutaneous connective tissue. It is widely utilised in cytotoxicity testing (*e.g.*, ISO 10993-5) due to its robust growth, consistent responses, and sensitivity to a broad range of toxic agents. Including both cell lines allows for a comprehensive assessment of tissue-specific *versus* general toxicological effects.

The MTT assay revealed distinct cytotoxic profiles across formulations and cell types. SP1 was the most cytotoxic among the tested Co(iii)PPIX nanoparticle formulations, with Caco-2 cells displaying marked sensitivity at concentrations up to 1.25 μg mL^−1^. The lowest viability for Caco-2 cells was recorded for SP1 at the lowest concentration tested (52.70%).

It suggests potential risks associated with intestinal exposure to this formulation. The pronounced cytotoxicity of SP1 may be linked to its low antioxidant capacity, as indicated by minimal DPPH radical scavenging activity (8.18%). The lack of effective antioxidant defence could increase oxidative stress in exposed cells, contributing to higher toxicity. Interestingly, despite its low antioxidant potential, SP1 was associated with increased Caco-2 cell viability at higher concentrations. This seemingly paradoxical effect may be explained by the reduced cellular uptake of larger particles, which limits intracellular stress responses. SP1 had one of the largest hydrodynamic diameters, and decreased internalisation at elevated concentrations could prevent the triggering of pro-apoptotic pathways, allowing for enhanced cell survival. Additionally, forming aggregates or a protein corona at higher doses may further limit direct nanoparticle–cell interactions, providing a protective effect. In this context, although coupled with weaker antioxidant properties, the large particle size may act as a passive modulator of cytotoxicity, indirectly supporting cell viability in Caco-2 cells.

For Caco-2 cells, a general trend of increased cell viability was observed at higher nanoparticle concentrations, especially for SP1 and SP7, which exhibited the largest hydrodynamic diameters. Cell viability values exceeded 100% in several cases, indicating improved survival relative to untreated control cells. In contrast to SP1, SP4 exhibited the most stable response, with only a 29.19% difference between its minimum and maximum viability. Overall, samples with smaller particle sizes tended to induce lower viability at intermediate concentrations, with recovery or improved viability at higher doses, a pattern consistent across multiple formulations.

In contrast, L929 cells showed a different viability profile. The highest viability was observed for SP1 at intermediate concentrations, but high concentrations led to greater mortality, in contrast to the trend seen in Caco-2 cells. SP2 behaved similarly in both lines, showing improved viability at higher concentrations. SP3 and SP5–SP7 shared a typical pattern of highest cytotoxicity at medium concentrations, while SP4 showed consistent viability across all tested doses.

Several possible mechanisms underlie the cytotoxicity of nanoparticles. Nanoparticles can directly damage cells by penetrating and interacting with intracellular components or indirectly by generating radicals and reactive oxygen species (ROS).^54^ Co(iii)PPIX demonstrates cytotoxic properties toward Caco-2 and L929 cell lines. One potential mechanism for the cytotoxicity of PPIX is the elevation of intracellular free heme levels, which is associated with increased ROS production.^55^ The production of ROS by heme occurs through the oxidation of Fe(ii) to Fe(iii); subsequently, hydrogen peroxide can cleave the heme ring, releasing redox-active iron that enhances the production of reactive oxygen species.^56^ ROS production by iron damages lipids, proteins, and DNA, ultimately resulting in cell death.^56,57^ PPIX is an inducer of heme oxygenase (HO). HO-1 is an enzyme released by cellular stressors that prevents oxidative DNA damage following exposure to reactive oxygen species.^58^ Busserolles *et al.* reported that CoPPIX administration reduced apoptosis in Caco-2 cancer cells induced by serum deprivation.^59^ Hirai *et al.* reported that CoPPIX reduced apoptosis in cancer cells exposed to nitric oxide.^60^ It may explain why, in our experiments, higher concentrations of Co(iii)PPIX nanoparticles were associated with greater Caco-2 cell survival, even exceeding control levels despite their inherently low antioxidant activity and cytotoxic behaviour at lower concentrations.

### Antibacterial activity

Metalloporphyrins can enter bacterial cells by utilising heme receptors whose expression depends on iron availability. The highest expression is observed in bacteria with limited access to iron. Consequently, metalloporphyrins are most active against bacteria cultured under low-iron conditions.^61^ A potential antibacterial mechanism of protoporphyrin involves the production of oxidative radicals or may affect the cells' ability to combat oxidative damage. It is supported by the fact that metalloporphyrins have proven ineffective against bacteria cultured in anaerobic conditions.^61^ Among metalloporphyrins, GaPPIX is considered one of the most potent antibacterial agents. Its close structural similarity to heme allows it to interfere with heme-dependent cytochromes, disrupting bacterial respiration.^62^ In contrast, heme, or iron protoporphyrin IX, exhibits antibacterial activity through oxidative reactions and only in the presence of reduced thiol reagents in the growth medium; bacteria resisted heme.^63^

**Fig. 9:**
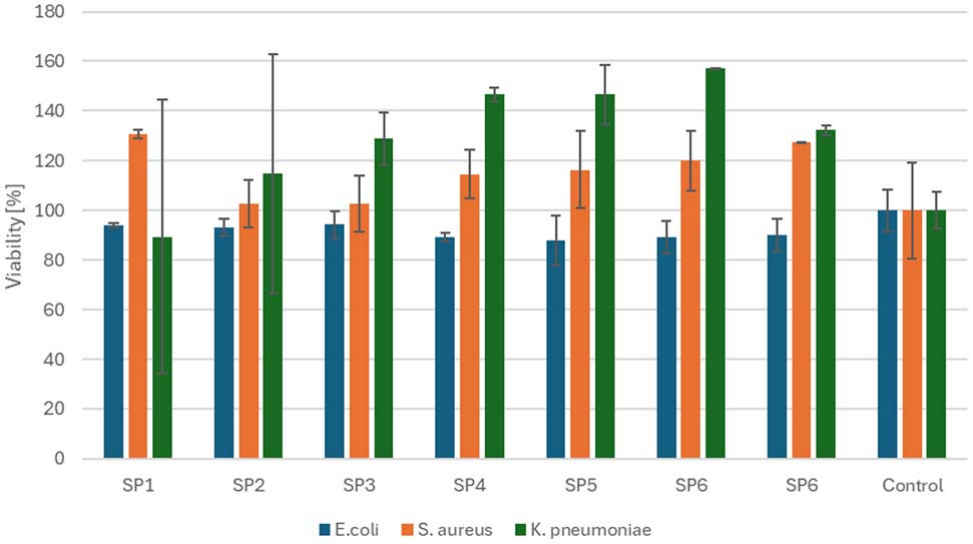
Antibacterial activity test results for CoPPIX NPs.

The antibacterial properties of Co(iii)PPIX nanoparticles were evaluated against three bacterial strains – *Escherichia coli*, *Staphylococcus aureus*, and *Klebsiella pneumoniae*, using resazurin fluorescence measurements (Fig. 9). CoPPIX NPs exhibit antibacterial properties against *E. coli*, with activity ranging from 5.66% (SP3) to 12.05% (SP5). For *S. aureus*, higher fluorescence values were recorded compared to the control, with the highest results for SP1 and SP7. Against *K. pneumoniae*, fluorescence values were lower than the control only for SP1 and SP2, with activity of 10.59% and 10.26%, respectively. These findings demonstrate that Co(iii)PPIX nanoparticles demonstrated low activity and only against the *E. coli* strain. It aligns with literature reports indicating a lack of CoPPIX activity against *S. aureus*, with the only PPIX variants exhibiting activity being FePPIX, GaPPIX, MnPPIX, and ZnPPIX.^61,64^ Literature reports suggest that cobalt nanoparticles exhibit more significant activity against Gram-negative bacteria than Gram-positive ones.^65,66^

### Distribution of CoPPIX nanoparticles in retentate and permeate after ultrafiltration

**Fig. 10:**
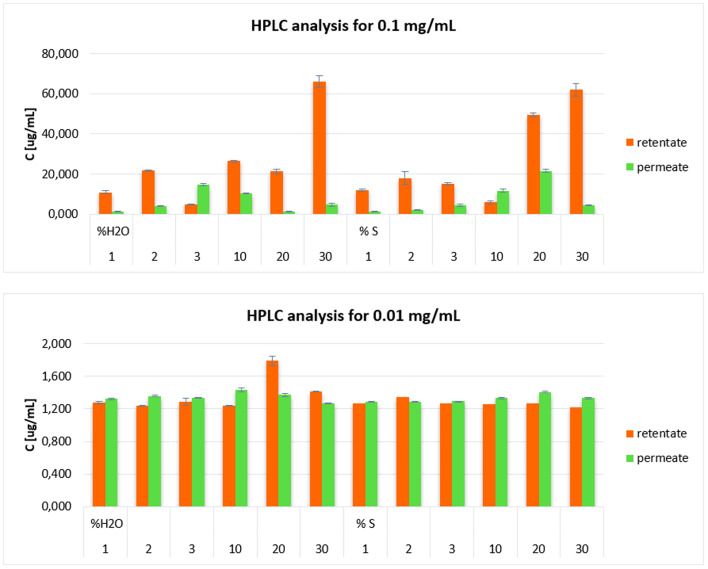
Fractionation of CoPPIX nanoparticles into retentate and permeate.

The concentrations of cobalt protoporphyrin IX (Co(iii)PPIX) nanoparticles in the permeate and retentate fractions obtained during the ultrafiltration (UF) process were determined by HPLC-MS/MS analysis. Quantification was performed for samples initially prepared at concentrations of 0.1 mg mL^−1^, 0.01 mg mL^−1^, and 0.001 mg mL^−1^. Results for 0.1 mg mL^−1^ and 0.01 mg mL^−1^ are presented in Fig. 10, whereas for the lowest concentration (0.001 mg mL^−1^), the nanoparticle levels were below the method's detection limit.

The UF process was applied to fractionate Co(iii)PPIX nanoparticles based on their size and colloidal stability using a membrane with a 3000 Da molecular weight cutoff. Fig. 10 illustrates the distribution of nanoparticles between the permeate and retentate phases under varying solvent conditions (water [W] or saline [S]) at two Co(iii)PPIX concentrations (0.1 mg mL^−1^ and 0.01 mg mL^−1^). Distinct distribution trends were observed depending on the solvent type and concentration, directly correlating with the hydrodynamic size and zeta potential values obtained from DLS measurements (Fig. 2). At an initial concentration of 0.1 mg mL^−1^, the highest Co(iii)PPIX nanoparticle concentration in the retentate was recorded for samples with 20% and 30% saline (S), followed by 30% water (W). This behaviour indicates significant nanoparticle aggregation, resulting in particle sizes exceeding the membrane's cutoff, thereby preventing permeation. These findings are consistent with DLS data (Fig. 2A), where the largest hydrodynamic diameter (1385 nm) was observed for the 20% S sample, with an overall trend of increasing particle size with higher solvent content.

Notably, a consistent trend was observed for both water and saline systems: the concentration of nanoparticles in the retentate progressively increased with increasing solvent percentage. The highest retentate concentrations were observed at 30% W and 20–30% S (Fig. 10). This trend was mirrored by the DLS measurements (Fig. 2A), where hydrodynamic diameter similarly increased with rising solvent content. Therefore, DLS results strongly correlate with the nanoparticle retention patterns determined by HPLC-MS/MS, confirming that the largest particle sizes coincided with the highest nanoparticle concentrations at 30% W and 20–30% S.

In contrast, the highest Co(iii)PPIX nanoparticle concentrations in the permeate at 0.1 mg mL^−1^ were observed for samples containing 10% and 20% S, as well as 3% and 10% W. These samples exhibited relatively smaller particle sizes and higher zeta potential values, favouring nanoparticle stability and membrane permeation.

According to the hydrodynamic size and zeta potential data (Fig. 2A), higher zeta potential values were associated with lower hydrodynamic diameters, indicating improved colloidal stability. Notably, samples with lower antisolvent content (1–3% W and S) demonstrated higher zeta potentials (*e.g.*, 17.7 mV for 2% S), which reduced particle aggregation and enhanced membrane permeation. In contrast, samples with high salt concentrations (10–30% S) showed a significant reduction in zeta potential, attributable to electrostatic screening effects. This decrease in surface charge promoted nanoparticle aggregation, leading to greater retention in the retentate. These observations are consistent with previous reports, demonstrating that lower zeta potential values are associated with increased particle size, reduced colloidal stability, and altered nanoparticle distribution between the permeate and retentate fractions.^67^

At an initial concentration of 0.01 mg mL^−1^, a slightly different distribution pattern was observed, characterised by lower nanoparticle concentrations in both permeate and retentate fractions. The highest nanoparticle concentration in the retentate was recorded for the sample with 20% W, reaching 1.787 μg mL^−1^. In other samples, the nanoparticle concentrations in the retentate remained relatively consistent regardless of whether water or saline was used. Similarly, the nanoparticle concentrations oscillated at comparable levels across all samples in the permeate, with no significant differences between water- and saline-based systems.

These results are consistent with the hydrodynamic size (DLS) and zeta potential measurements (Fig. 2B). The sample containing 20% W exhibited a larger hydrodynamic diameter, suggesting an enhanced tendency for aggregation and thus a higher degree of retention in the retentate. In contrast, samples with lower antisolvent contents (1–3%) demonstrated moderate particle sizes and relatively higher zeta potentials, indicating better colloidal stability and reduced aggregation, limiting nanoparticle retention.

Lower antisolvent concentrations led to higher zeta potential values, enhancing nanoparticle electrostatic repulsion and preventing aggregation. Conversely, higher antisolvent concentrations, especially in saline systems, caused a significant decrease in zeta potential due to electrostatic screening, thereby promoting aggregation.^40,68^

HPLC-MS/MS results for the nanoparticle distribution at 0.01 mg mL^−1^ are strongly correlated with the DLS and zeta potential data: samples characterised by larger particle sizes and lower zeta potentials (*e.g.*, 20% W) showed increased retention, whereas more stable and smaller-sized nanoparticles with higher zeta potentials demonstrated enhanced permeation across the membrane.

## Conclusions

Co(iii)PPIX nanoparticles were obtained by adding an antisolvent to the Co(iii)PPIX solution, followed by UF to investigate the influence of non-NP-forming particles on the physicochemical and biological properties of Co(iii)PPIX NPs. The UF process allowed NPs to be produced with smaller hydrodynamic sizes and lower zeta potentials. UF also positively impacted the UV-Vis spectrum. In the case of Raman spectroscopy, slight differences were observed between the spectra of UF-treated samples and those without UF, as well as among the various substrates used. Biological studies revealed that Co(iii)PPIX nanoparticles exhibit free radical scavenging activity, with antioxidant intensity correlating inversely with particle size. In studies on cell lines, cytotoxic properties of the tested particles were noted at low applied concentrations.

In contrast, increased cell survival was observed at higher concentrations, sometimes exceeding the survival rates of control samples. CoPPIX is a known inducer of heme oxygenase-1 (HO-1). HO-1 protects healthy tissues from the induction of some types of cancers. However, if, despite this protection, the disease begins to develop, HO-1 begins to protect cancer cells, improving their survival and resistance to treatment. However, it should be kept in mind that many of the pharmacological effects of HO-1 inhibitors and HO-1 activators (*e.g.* CoPPIX) are independent of HO-1 because all of these compounds exhibit strong, nonspecific functions. In monocyte heme, THP-1, ZnMPIX, ZnDPPIX, and CoPPIX, regardless of their effect on HO-1, reduced IFN-γ signal transduction, while SnPPIX enhanced it and increased MHC-II expression. CoPPIX, SnPPIX, and ZnPPIX are direct inhibitors of caspase-3 and caspase-8 and thus mitigate the apoptosis rate independently of the HO-1 pathway.^69^ In antibacterial studies, Co(iii)PPIX nanoparticles exhibited selective activity against *Escherichia coli*. In conclusion, this work is multifunctional in design, encompassing the synthesis of Co(iii)PPIX NPs, the investigation of their physicochemical properties and biological activities, similar to the recent work by Khalid *et al.* on silver nanoparticles,^70^ and the resulting NPs reflect trends observed in silver nanoparticles modified with biopolymers.^71^

While these findings demonstrate promising multifunctional activity, there is a further need for in-depth mechanistic studies of the observed biological activity at the molecular level and further studies of nanoparticles' long-term stability and action *in vivo*.

## Conflicts of interest

There are no conflicts to declare.

## Supplementary Material

RA-015-D5RA07110K-s001

## Data Availability

All data needed to evaluate the conclusions in the paper are present in the paper or the SI. Supplementary information: the results of the MTT test on cell lines and positive-ion ESI mass spectrum of cobalt protoporphyrin IX. See DOI: https://doi.org/10.1039/d5ra07110k.

## References

[cit1] Imran M., Ramzan M., Qureshi A. K., Azhar Khan M., Tariq M. (2018). Biosensors.

[cit2] Karl M. K. , Kevin M. S. and Roger G., The Porphyrin Handbook, Inorganic, Organometallic and Coordination Chemistry, Academic Press, 3, 2000, 325

[cit3] Cheng W., Haedicke I. E., Nofiele J., Martinez F., Beera K., Scholl T. J., Cheng H. L. M., Zhang X. A. (2014). J. Med. Chem..

[cit4] Venter A., Szulc D. A., Loai S., Ganesh T., Haedicke I. E., Cheng H. L. M. (2018). Sci. Rep..

[cit5] Zhang Q., He J., Yu W., Li Y., Liu Z., Zhou B., Liu Y. (2020). RSC Med. Chem..

[cit6] Chen J., Chen F., Zhang L., Yang Z., Deng T., Zhao Y., Zheng T., Gan X., Zhong H., Geng Y., Fu X., Wang Y., Yu C. (2021). ACS Appl. Mater. Interfaces.

[cit7] Costa e Silva R., da Silva L. O., de Andrade Bartolomeu A., Brocksom T. J., de Oliveira K. T. (2020). Beilstein J. Org. Chem..

[cit8] Sachar M., Anderson K. E., Ma X. (2016). J. Pharmacol. Exp. Ther..

[cit9] Xu H., Sun Y., Zhang Y., Wang W., Dan J., Yao J., Chen H., Tian F., Sun X., Guo S., Tian Z., Tian Y. (2014). Cell. Physiol. Biochem..

[cit10] Shan Y., Lambrecht R. W., Donohue S. E., Bonkovsky H. L., Shan Y., Lambrecht R. W., Donohue S. E., Bonkovsky H. L. (2006). Faseb. J..

[cit11] Seiwert N., Wecklein S., Demuth P., Hasselwander S., Kemper T. A., Schwerdtle T., Brunner T., Fahrer J. (2020). Cell Death Disease.

[cit12] Szade A., Szade K., Nowak W. N., Bukowska-Strakova K., Muchova L., Gońka M., Żukowska M., Cieśla M., Kachamakova-Trojanowska N., Rams-Baron M., Ratuszna A., Dulak J., Józkowicz A. (2019). EMBO Mol. Med..

[cit13] Siddique A. B., Shaheen M. A., Abbas A., Zaman Y., Rasheed M. U., Karim A., Mustaqeem M., ur Rehman M. F., Alam M. M., Alahmari A. S. (2025). Water, Air, Soil Pollut..

[cit14] Kanwal M., Sher M., Abbas A., Akhtar S., Siddique A. B., ul Hasan M. N., Assad N., Alhazmi H. A., Amin H. M. A. (2025). J. Water Proc. engineering.

[cit15] Jabbar A., Abbas A., Assad N., Naeem-ul-Hassan M., Alhazmi H. A., Najmi A., Zoghebi K., Al Bratty M., Hanbashi A., Amin H. M. A. (2023). RSC Adv..

[cit16] Makhadmeh G. N., Abdul Aziz A. (2018). Artif. Cells, Nanomed. Biotechnol..

[cit17] da Silva D. B., da Silva C. L., Davanzo N. N., da Silva Souza R., Correa R. J., Tedesco A. C., Riemma Pierre M. B. (2021). Photodiagnosis Photodyn. Ther..

[cit18] Ning L. G., Liu P., Wang B., Li C. M., Kang E. T., Lu Z. S., Hu X. F., Xu L. Q. (2019). J. Colloid Interface Sci..

[cit19] Rouhani H., Sepehri N., Montazeri H., Khoshayand M. R., Ghahremani M. H., Ostad S. N., Atyabi F., Dinarvand R. (2014). Pharm. Res..

[cit20] Yao M., Ma M., Zhang H., Zhang Y., Wan G., Shen J., Chen H., Wu R. (2018). Adv. Funct. Mater..

[cit21] Liu Y., Yan S., Li M., Wang K., Zeng D., Yang H. (2019). Colloids Surf., A.

[cit22] Kennedy J. C., Pottier R. H. (1992). J. Photochem. Photobiol. B Biol..

[cit23] Schneppensieper T., Zahl A., van Eldik R. (2001). Angew. Chem., Int. Ed..

[cit24] Wolak M., van Eldik R. (2005). J. Am. Chem. Soc..

[cit25] Jee J. E., Wolak M., Balbinot D., Jux N., Zahl A., van Eldik R. (2006). Inorg. Chem..

[cit26] Jee J. E., Eigler S., Hampel F., Jux N., Wolak M., Zahl A., Stochel G., van Eldik R. (2005). Inorg. Chem..

[cit27] Jee J. E., Eigler S., Jux N., Zahl A., van Eldik R. (2007). Inorg. Chem..

[cit28] Franke A., Stochel G., Jung C., van Eldik R. (2004). J. Am. Chem. Soc..

[cit29] Wolak M., Zahl A., Schneppensieper T., Stochel G., van Eldik R. (2001). J. Am. Chem. Soc..

[cit30] Wolak M., Stochel G., Hamza M., van Eldik R. (2000). Inorg. Chem..

[cit31] Wolak M., van Eldik R. (2007). Chem.–Eur. J..

[cit32] Franke A., Wolak M., van Eldik R. (2009). Chem.–Eur. J..

[cit33] Oszajca M., Drzewiecka-Matuszek A., Franke A., Rutkowska-Zbik D., Brindell M., Witko M., Stochel G., van Eldik R. (2014). Chem.–Eur. J..

[cit34] Franke A., van Eldik R. (2015). Chem.–Eur. J..

[cit35] Wu C. Y., Wang W. (2022). Pharmaceutics.

[cit36] Siddique A. B., Shaheen M. A., Abbas A., Zaman Y., Amin H. M. A., Alam M. M., Alharbi N. K., Alshehri F., Shami A., Al-Joufi F. A., Ali A. (2025). Int. J. Environ. Anal. Chem..

[cit37] Wang L. Y., Wu M. Y., Wu Z. Y., Li Y. T. (2025). J. Mol. Struct..

[cit38] Zeng G., Wu Z., Cao W., Wang Y., Deng X., Zhou Y. (2018). Nat. Prod. Res..

[cit39] Ahmadi R., Hosseini H. R. M., Masoudi A., Omid H., Namivandi-Zangeneh R., Ahmadi M., Ahmadi Z., Gu N. (2013). Colloids Surf. A Physicochem. Eng. Asp..

[cit40] Ahmed R., ul ain Hira N., Fu Z., Wang M., Halepoto A., Khanal S., Iqbal S., Mahar H., Cohen Stuart M. A., Guo X. (2021). ACS Omega.

[cit41] El-Demrdash S. A., Nixon-Luke R., Thomsen L., Tadich A., Lau D. W. M., Chang S. L. Y., Greaves T. L., Bryant G., Reineck P. (2021). Nanoscale.

[cit42] Gordillo-Galeano A., Mora-Huertas C. E. (2021). Colloids Surf. A Physicochem. Eng. Asp..

[cit43] Myrzakhmetov B., Arnoux P., Mordon S., Acherar S., Tsoy I., Frochot C. (2021). Pharmaceuticals.

[cit44] Kim B. F., Bohandy J. (1981). Johns Hopkins APL Tech. Dig..

[cit45] Kadiyala N. K., Mandal B. K., Ranjan S., Dasgupta N. (2018). Mater. Sci. Eng., C.

[cit46] Akhtar K. , Khan S. A., Khan S. B. and Asiri A. M., Handbook of Materials Characterization, Springer Cham., 2018, 236

[cit47] Su Y., Zhu H., Dong H. (2014). Anal. Lett..

[cit48] Roslan M. S., Chaudary K. T., Haider Z., Zin A. F. M., Ali J. (2017). AIP Conf. Proc..

[cit49] Carvalho I. M. M., Ogawa M. Y. (2010). J. Braz. Chem. Soc..

[cit50] Dinache A., Nistorescu S., Tozar T., Smarandache A., Boni M., Prepelita P., Staicu A. (2023). Molecules.

[cit51] Sun Z. C., Bin She Y., Zhou Y., Song X. F., Li K. (2011). Molecules.

[cit52] Martínez A., López-Rull I., Fargallo J. A. (2023). Antioxidants.

[cit53] Marin-Flores C. A., Rodríguez-Nava O., García-Hernández M., Ruiz-Guerrero R., Juárez-López F., Morales-Ramírez A. de J. (2021). J. Mater. Res. Tech..

[cit54] Abbasi R., Shineh G., Mobaraki M., Doughty S., Tayebi L. (2023). J. Nanopart. Res..

[cit55] Zhang Y., Zhang X., Zhou B. (2023). Antioxidants.

[cit56] Belcher J. D., Beckman J. D., Balla G., Balla J., Vercellotti G. (2010). Antioxid. Redox Signaling.

[cit57] Kumar S., Bandyopadhyay U. (2005). Toxicol. Lett..

[cit58] Batista S., Bocanegra-Becerra J. E., Claassen B., Rubião F., Rabelo N. N., Figueiredo E. G., Oberman D. Z. (2023). WorldNSX.

[cit59] Busserolles J., Megías J., Terencio M. C., Alcaraz M. J. (2006). Int. J. Biochem. Cell Biol..

[cit60] Hirai K., Sasahira T., Ohmori H., Fujii K., Kuniyasu H. (2006). Int. J. Cancer.

[cit61] Stojiljkovic I., Kumar V., Srinivasan N. (1999). Mol. Microbiol..

[cit62] Hijazi S., Visca P., Frangipani E. (2017). Front. Cell. Infect. Microbiol..

[cit63] Nitzan Y., Ladan H., Gozansky S., Malik Z. (1987). FEMS Microbiol. Lett..

[cit64] Wakeman C. A., Stauff D. L., Zhang Y., Skaar E. P. (2014). J. Bacteriol..

[cit65] Abass A. A., Abdulridha W. M., Alaarage W. K., Abdulrudha N. H., Haider J. (2021). J. Med. Life.

[cit66] Raja F. N. S., Worthington T., Martin R. A. (2023). Biomed. Mater..

[cit67] Ceballos-Chuc M. C., Ramos-Castillo C. M., Rodríguez-Pérez M., Ruiz-Gómez M. Á., Rodríguez-Gattorno G., Villanueva-Cab J. (2022). Inorganics.

[cit68] Schroën K., van Dinther A., Stockmann R. (2017). Separ. Purif. Technol..

[cit69] Jozkowicz A., Was H., Dulak J. (2007). Antioxid. Redox Signaling.

[cit70] Khalid Z., Ali A., Siddique A. B., Zaman Y., Sibtain M. F., Abbas A., Alam M. M., Alwethaynani M. S. (2025). RSC Adv..

[cit71] Siddique A. B., Amr D., Abbas A., Zohra L., Irfan M. I., Alhoshani A., Ashraf S., Amin H. M. A. (2024). Int. J. Biol. Macromol..

