# Supplementary material for: Synthesis of nanoparticles of cobalt protoporphyrin IX (Co(iii)PPIX NPs). Antiradical, cytotoxicity and antibacterial properties
Source: RSC Adv. 2025 Oct 3;15(44):36789–802. doi: 10.1039/d5ra07110k (PMC12495410; doi:10.1039/d5ra07110k)
Supplement: RA-015-D5RA07110K-s001 [file RA-015-D5RA07110K-s001.pdf]

Supplementary Information (SI) for RSC Advances.
This journal is © The Royal Society of Chemistry 2025

Synthesis of nanoparticles of cobalt protoporphyrin IX (Co(III)PPIX NPs). Antiradical, cytotoxicity and antibacterial properties

Piotr Fijałkowski [a], Olga Impert [b*], Paweł Pomastowski [a], Katarzyna Rafińska [a,c], Justyna Walczak-Skierska [a], Paweł Fijałkowski [a,c], Anna Katafias [b*] and Rudi van Eldik [b,d]

[a] Centre for Modern Interdisciplinary Technologies, Nicolaus Copernicus University in Toruń, Wilenska 4, 87-100 Toruń, Poland

[b] Department of Inorganic and Coordination Chemistry, Faculty of Chemistry, Nicolaus Copernicus University in Toruń, Gagarina 7, 87-100 Toruń, Poland

[c] Department of Environmental Chemistry and Bioanalytics, Faculty of Chemistry, Nicolaus Copernicus University in Toruń, Gagarina 7, Toruń 87-100, Poland

[d] Department of Chemistry and Pharmacy, University of Erlangen-Nuremberg, Egerlandstrasse 1, 91058 Erlangen, Germany

*corresponding authors: oimpert@umk.pl (Olga Impert), katafias@umk.pl (Anna Katafias)

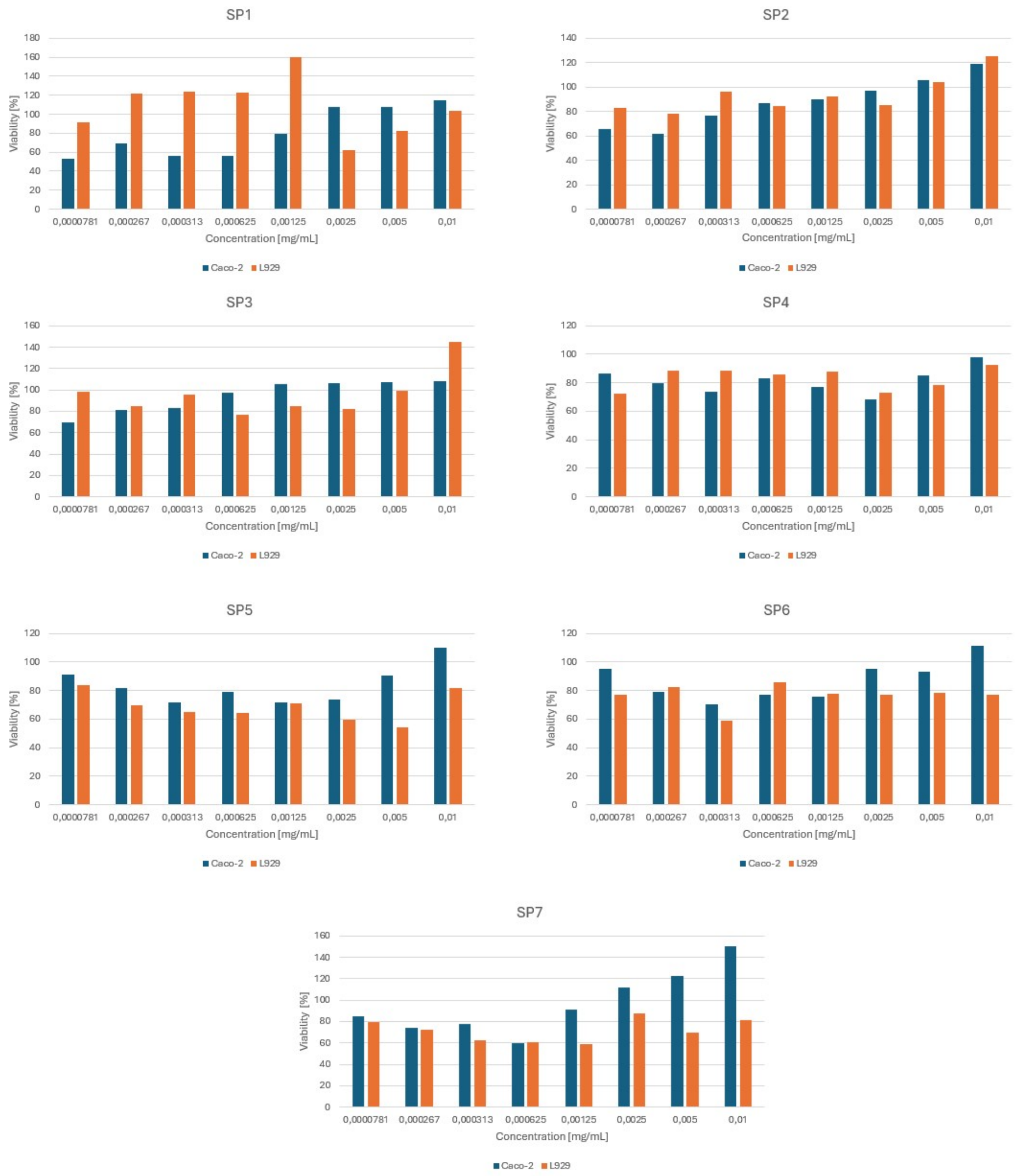

Figure S.1. Results of Co(III)PPIX Np's MTT cytotoxicity assay on Caco-2 (blue) and L929 (red) cell lines.

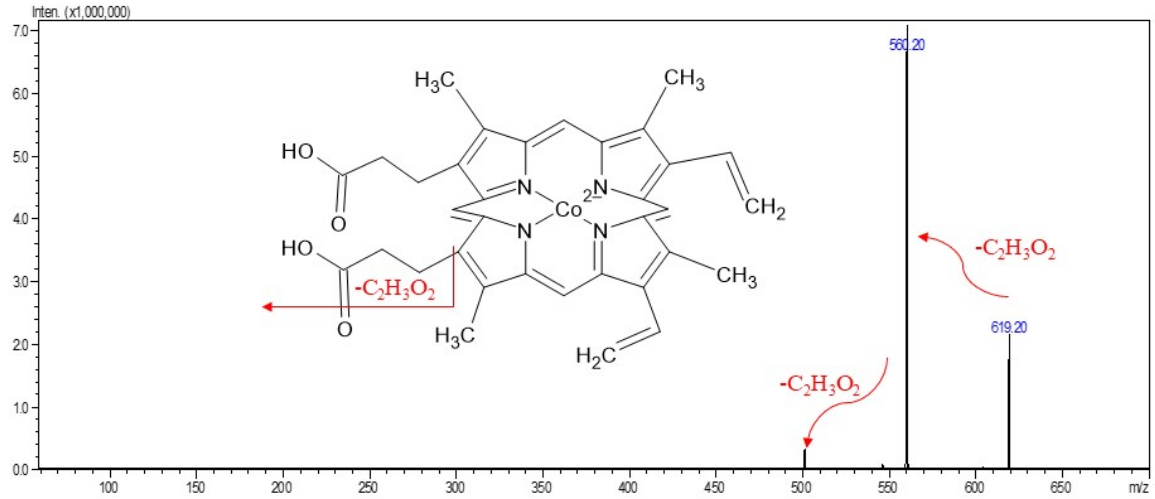

Figure S.2. Positive-ion ESI mass spectrum of cobalt protoporphyrin IX. Loss od carboxylic acid groups as indicated in the MS/MS spectra, fragment ions with *m/z* 560 and 501.
